# Advancements in Personalized CAR-T Therapy: Comprehensive Overview of Biomarkers and Therapeutic Targets in Hematological Malignancies

**DOI:** 10.3390/ijms25147743

**Published:** 2024-07-15

**Authors:** Wioletta Olejarz, Karol Sadowski, Daniel Szulczyk, Grzegorz Basak

**Affiliations:** 1 Department of Biochemistry and Pharmacogenomics, Faculty of Pharmacy, Medical University of Warsaw, 02-097 Warsaw, Poland; karol.sadowski@wum.edu.pl; 2 Centre for Preclinical Research, Medical University of Warsaw, 02-097 Warsaw, Poland; 3 Department of Hematology, Transplantation and Internal Medicine, Medical University of Warsaw, 02-097 Warsaw, Poland; grzegorz.basak@wum.edu.pl; 4 Chair and Department of Biochemistry, The Medical University of Warsaw, 02-097 Warsaw, Poland; daniel.szulczyk@wum.edu.pl

**Keywords:** CAR-T, EVs, immunotherapy, biomarkers, hematological malignancies, checkpoint inhibitors, TME

## Abstract

Chimeric antigen receptor T-cell (CAR-T) therapy is a novel anticancer therapy using autologous or allogeneic T-cells. To date, six CAR-T therapies for specific B-cell acute lymphoblastic leukemia (B-ALL), non-Hodgkin lymphomas (NHL), and multiple myeloma (MM) have been approved by the Food and Drug Administration (FDA). Significant barriers to the effectiveness of CAR-T therapy include cytokine release syndrome (CRS), neurotoxicity in the case of Allogeneic Stem Cell Transplantation (Allo-SCT) graft-versus-host-disease (GVHD), antigen escape, modest antitumor activity, restricted trafficking, limited persistence, the immunosuppressive microenvironment, and senescence and exhaustion of CAR-Ts. Furthermore, cancer drug resistance remains a major problem in clinical practice. CAR-T therapy, in combination with checkpoint blockades and bispecific T-cell engagers (BiTEs) or other drugs, appears to be an appealing anticancer strategy. Many of these agents have shown impressive results, combining efficacy with tolerability. Biomarkers like extracellular vesicles (EVs), cell-free DNA (cfDNA), circulating tumor (ctDNA) and miRNAs may play an important role in toxicity, relapse assessment, and efficacy prediction, and can be implicated in clinical applications of CAR-T therapy and in establishing safe and efficacious personalized medicine. However, further research is required to fully comprehend the particular side effects of immunomodulation, to ascertain the best order and combination of this medication with conventional chemotherapy and targeted therapies, and to find reliable predictive biomarkers.

## 1. Introduction

CAR-T therapy, an innovative anticancer treatment, utilizes engineered allogeneic or autologous T-cells to express a chimeric antigen receptor (CAR) targeting a membrane antigen [1,2]. CARs are synthetic receptors engineered to redirect lymphocytes, typically T-cells, toward identifying and removing cells that express a particular target antigen. Unlike T-cells, CAR-Ts have the ability to recognize antigens present on cancer cells’ surfaces independently of human major histocompatibility complex (MHC) molecules [3]. The first engineered T-cell with a chimeric molecule was created in 1993 by Israeli immunologist Zelig Eshhar. Since then, numerous modifications have been made, including the incorporation of a co-stimulatory domain to enhance the antitumor potency of CAR-Ts. The initial clinical application of CAR-Ts occurred in Rotterdam in 2005 for metastatic renal cell carcinoma and simultaneously at the National Cancer Institute (NCI) for metastatic ovarian cancer. Significant clinical success was achieved with anti-CD19 CAR-Ts, first used in 2009 by Steven Rosenberg at the NCI in a patient with refractory follicular lymphoma (FL), and later in 2011 by Carl June and David Porter from the University of Pennsylvania in patients with chronic lymphocytic leukemia (CLL) and B-cell acute lymphoblastic leukemia (B-ALL). Since then, major centers in North America have initiated numerous early phase and pivotal trials, demonstrating unprecedented response rates in heavily pretreated, chemorefractory patients with B-cell malignancies. These clinical successes led to the approval of three anti-CD19 CAR-T products for the treatment of B-cell malignancies in the United States and Europe as of December 2020 [4].

In Europe, Tisagenlecleucel (Kymriah™) is approved for treating children and young adults with refractory/relapsed (r/r) acute lymphoblastic leukemia, as well as r/r diffuse large B-cell lymphoma and follicular lymphoma (FL). Additionally, axicabtagene ciloleucel (Yescarta™) is approved for adult patients with diffuse large B-cell lymphoma (DLBCL), high-grade B-cell lymphoma (HGBL), primary mediastinal large B-cell lymphoma (PMBCL), and r/r FL. The European Society of Blood and Marrow Transplantation has approved the preparation of these genetically modified autologous T-cells that specifically target CD19 and include guidelines [5,6]. To this date, the Food and Drug Administration (FDA) has approved six CAR-T therapies for specific B cell acute lymphoblastic leukemia (B-ALL), non-Hodgkin lymphomas (NHL), and multiple myeloma [7,8,9].

Significant barriers to the effectiveness of CAR-T therapy include cytokine release syndrome (CRS) [10], neurotoxicity [11], graft-versus-host-disease (GVHD) in the case of Allogeneic Stem Cell Transplantation (Allo-SCT), antigen escape, modest antitumor activity, restricted trafficking, limited persistence, the immunosuppressive microenvironment , and senescence and exhaustion of CAR-Ts [12]. Moreover, a significant challenge in the clinical management of these cancer patients continues to be cancer therapy resistance [13,14,15]. The pre-infusion immunological status of the patients has also impacted the effectiveness of CAR-T infusion. One factor is the enumeration of circulating monocytes and a monocyte gene signature in leukapheresis products, which can identify patients at very high risk of progression after CAR-T therapy [16]. It was also shown that the composition of the types of lymphocyte populations before CAR-T infusions is associated with the occurrence of ICANS [17]. Advances in the design and manufacture of monoclonal antibodies, antibody–drug conjugates, and bispecific T-cell engagers make the agents more powerful with fewer toxicities [18]. Additionally, immune checkpoint inhibitors (ICIs) mitigate the inhibition of immune regulatory mechanisms, resulting in the immunoablation of extremely resistance cancers [19]. The antibodies against programmed cell death protein 1 (PD-1) (pembrolizumab, nivolumab, cemiplimab), and its ligand, PD-L1 (atezolizumab, avelumab, and durvalumab), have been the main focus of the current clinical use of checkpoint inhibitors [20]. PD-1, CTLA-4, lymphocyte activation gene-3 (LAG-3), and mucin-domain containing-3 (TIM-3) are T-cell exhaustion markers that function as co-inhibitory receptors with a significant role in regulating T-cell responses in hematological malignancies [18,21]. Recent progress in the advancement of reasonable combinations of targeted approaches significantly improved therapeutic effects in hematological malignancies [22]. Importantly, patients with relapsed or refractory Hodgkin lymphoma (HL) showed a high rate of durable responses with an excellent safety profile following treatment with CD30-specific CAR-Ts (CD30.CAR-T). This highlights the potential of extending CAR-T therapies beyond canonical B-cell malignancies [23,24]. However, the overall clinical response rate to tumor immunotherapy still requires enhancement, underscoring the need for identifying new biomarkers and advancing therapeutic agents to achieve a more effective antitumor response [25].

This review aims to comprehensively characterize the ALL, NHL, and MM immune landscape, deciphering the differential roles of CAR-T receptors and checkpoint components in drug resistance, and suggests targets and markers for combination immunotherapies.

## 2. Acute Lymphoblastic Leukemia (ALL)

Acute lymphoblastic leukemia represents the malignant transformation and uncontrolled proliferation of lymphoid progenitor cells. ALL development entails the atypical expansion and specialization of a clonal group of lymphoid cells [26]. Most clinical symptoms observed in ALL indicate inadequately differentiated lymphoid cells in the bone marrow and peripheral blood. The initial manifestations of ALL may lack specificity and often involve a blend of constitutional symptoms and indications of bone marrow dysfunction (such as anemia, thrombocytopenia, and leukopenia) [27,28]. The studies, including those involving children, showed that certain genetic syndromes (Down syndrome, Fanconi anemia, Bloom syndrome, ataxia–telangiectasia, and Nijmegen syndrome) have been identified as predisposing factors for a minority of ALL cases [29,30]. Other factors that increase susceptibility to ALL include exposure to ionizing radiation, pesticides, specific solvents, and certain viruses like the Epstein–Barr Virus and the Human Immunodeficiency Virus [31,32].

The presence of 20% or more lymphoblasts in either the peripheral blood or bone marrow confirms the ALL diagnosis. A range of diagnostic methods, including immunophenotyping, flow cytometry, morphological assessment, and cytogenetic testing, are useful in verifying the diagnosis and establishing risk classification. A complete blood count with differential and a smear to evaluate the coagulation process and various blood cell types are two further assessments [28].

The main treatment for ALL in adults is typically long-term chemotherapy. The other options comprise stem cell or bone marrow transplantation, steroids, growth factors, targeted cancer drugs, and immunotherapy like CAR-T therapy or radiotherapy [33]. The treatment protocol for ALL typically comprises three sequential stages. The first stage, known as remission induction, focuses on eradicating leukemia cells residing in the bone marrow, restoring the appropriate cellular composition of the blood, and alleviating associated symptoms. The second stage, consolidation therapy, aims to eliminate any residual leukemia cells that may remain. The third stage, maintenance therapy, involves administering regular doses of chemotherapy drugs intended to prevent the recurrence of leukemia. The initial treatment regimen commonly consists of vincristine, corticosteroids, and anthracycline [34]. Once a complete response is achieved, there are several treatment options available, including consolidation and maintenance chemotherapy, as well as Allo-SCT for eligible patients. Allo-SCT has traditionally been regarded as the standard of care and the most effective approach for obtaining a long-lasting response in high-risk patients and those with relapsed or refractory disease [35,36,37]. The ELIANA trial, a phase 2 study conducted at 25 sites, investigated CTL019 in pediatric and young adult patients with B-cell ALL, enrolling 75 participants. The overall remission rate within 3 months was 81%, as determined by negative MRD assessed via flow cytometry. The event-free survival (EFS) rates at 6 months and 12 months were 73% and 50%, respectively, while the overall survival (OS) rates at 6 months and 12 months were 90% and 76%. Based on these findings, the FDA approved tisagenlecleucel for treating patients up to 25 years old with refractory, secondary, or later relapsed B-ALL [38,39]. ZUMA-3 was a study that assessed brexucabtagene autoleucel (KTE-X19). In the phase 1 trial, the overall complete response (CR) or CR with incomplete hematologic recovery (CRi) rate was 83%. These findings were corroborated in the phase 2 cohort, which showed a CR/CRi rate of 71% (39 out of 55 patients) with a median follow-up of 16.4 months. The median durations of remission, relapse-free survival (RFS), and overall survival (OS) were 12.8 months, 11.6 months, and 18.2 months, respectively. Based on these outcomes, the FDA approved KTE-X19 for adult patients with relapsed and/or refractory B-cell ALL [40,41].

## 3. Non-Hodgkin Lymphoma (NHL)

Non-Hodgkin lymphoma includes a wide range of lymphomas. Approximately 85–90% originate from B cells, while the remaining lymphomas arise from T-cells or natural killer (NK) cells [42]. The most common one is diffuse large B-cell lymphoma (DLBCL) [43]. Immune suppression is widely recognized as the primary established risk factor for developing NHL. The Epstein–Barr virus is frequently linked to several B-cell lymphomas, such as Burkitt lymphoma [44,45].

Currently, the World Health Organization’s classification of lymphoid neoplasms is used in the NHL diagnostic process. The four main classification categories for lymphoid neoplasms are immunodeficiency-associated lymphoproliferative disorders, mature B-cell neoplasms, mature T-/NK-cell neoplasms, and precursor B- and T-cell neoplasms [46]. Obtaining an accurate lymphoma diagnosis is crucial as it plays a significant role in determining the appropriate treatment for the patient. Additionally, the management approach is influenced by the stage of the disease and the presence or absence of prognostic factors that indicate the likely outcome of the disease [47], establishing a diagnosis based on an appropriate biopsy sample thoroughly evaluated.

There are two prognosis groups for NHL: aggressive lymphomas and indolent lymphomas. Indolent NHL types generally have a favorable prognosis, with a median survival reaching up to 20 years. Radiation therapy alone can be an effective treatment approach for early-stage indolent NHL. On the other hand, aggressive NHL has a shorter median survival, but a significant proportion of patients can achieve a cure through intensive combination chemotherapy [48,49,50]. Three large-scale multicenter phase 3 clinical trials—ZUMA-7, TRANSFORM, and BELINDA—were conducted for patients with B-cell NHL, each featuring a distinct second-generation CAR construct. Both ZUMA-7 and BELINDA led to FDA approval for the CD19 CAR-T products axicabtagene ciloleucel (axi-cel) and tisagenlecleucel (tisa-cel), respectively [51,52,53,54].

## 4. Multiple Myeloma (MM)

Multiple myeloma is a blood cancer characterized by clonal expansion of transformed plasma cells. While long-term disease control is possible for many patients, tumor resistance usually develops, causing relapse. This is particularly common in patients with triple-class refractory MM, which is resistant to immunomodulatory agents, proteasome inhibitors, and monoclonal antibodies [55]. Therapies targeting B-cell maturation antigen (BCMA), such as bispecific antibodies (BsAbs) and antibody–drug conjugates (ADCs), show great promise in treating MM [56]. CAR-T therapy causes rapid, profound, and long-lasting responses in heavily pretreated MM patients, while maintaining a manageable safety profile [57,58,59].

Idecabtagene vicleucel (ide-cel) has been approved by the US FDA for the treatment of relapsed and refractory multiple myeloma (RRMM), making it the first CAR-T product approved for myeloma [60]. This therapy resulted in significant and long-lasting responses in patients with relapsed and refractory MM who have undergone extensive prior treatments [61]. A response occurred in 71% of patients in the ide-cel group and 42% of those in the standard-regiment group, while a complete response occurred in 39% and 5%, respectively [62].

Ciltacabtagene autoleucel (cilta-cel), a CAR-T therapy targeting the B-cell maturation antigen (BCMA), is effective in patients with relapsed or refractory MM who have undergone extensive previous treatments. In the cilta-cel group, more patients achieved an overall response (84.6% compared to 67.3% in the standard care group), a complete response or better (73.1% vs. 21.8%), and an absence of minimal residual disease (60.6% vs. 15.6%) [63].

## 5. Minimal/Measurable Residual Disease (MRD) in ALL, NHL, and MM

Minimal/measurable residual disease describes a population of leukemia cells that have survived chemotherapy or radiotherapy and can lead to recurrence of the disease [64]. Although over 75% of adult patients with ALL attain complete remission through intensive chemotherapy, approximately 40% of them relapse within five-year period, likely attributed to residual leukemic cells [65]. Molecular techniques for investigating MRD in ALL are polymerase chain reaction (PCR) amplification-based methods, which stand out as the most standardized approaches [66]. The advent of diagnostic platforms, such as next-generation sequencing (NGS), has brought about substantial progress in enhancing the sensitivity of MRD diagnostics [67]. It was shown that the ultrasensitive detection of residual and relapse clones that determine the MRD improves the complete remission cases [68]. Gene rearrangements serve as indicators of clonality, allowing for the highly sensitive detection of monoclonal leukemic lymphoid cells [69]. A comprehensive understanding of the genetic basis of ALL and improvements in evaluating treatment response via serial minimal residual disease (MRD) have led to a decrease in mortality rates for children diagnosed with ALL in the US [70,71]. Assessing the efficacy of treatment and predicting long-term prognosis in patients with B-cell non-Hodgkin lymphomas (B-NHL) is crucial through the use of minimal residual disease (MRD) diagnostics [72,73,74]. Detecting MRD through flow cytometry (FC) may improve assessment of response to therapy and prognostication of MM patients [75,76,77]. Importantly, next-generation flow (NGF) and next-generation sequencing (NGS), together with digital PCR (dPCR), mass spectrometry, and imaging techniques, have been developed to provide higher levels of sensitivity to detect MRD in MM [78,79,80].

## 6. Co-Stimulation and Co-Inhibition of CAR-Ts

**Figure 1:**
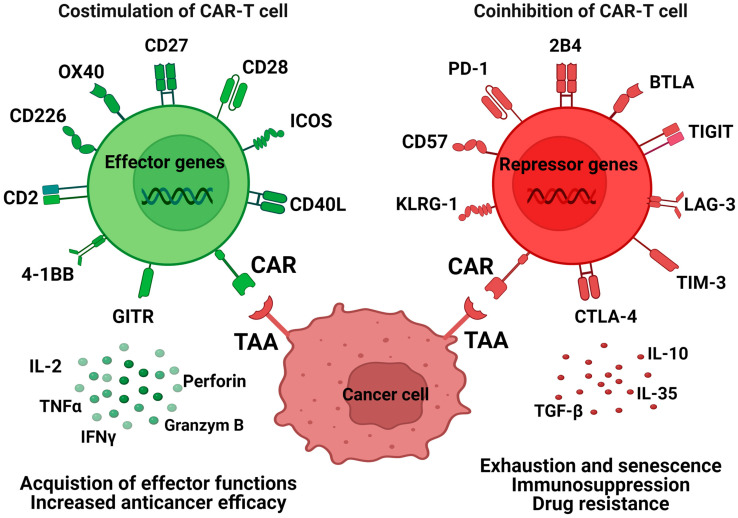
Co-stimulatory and co-inhibitory receptors of CAR-Ts [96], modified. B- and T-lymphocyte attenuator (BTLA), T-cell immunoreceptor with immunoglobulin and ITIM domain (TIGIT), lymphocyte-activated gene 3 (LAG-3), T-cell immunoglobulin and mucin domain 3 (TIM-3), cytotoxic T-lymphocyte-associated protein-4 (CTLA-4), programmed cell death protein 1 (PD-1), killer cell lectin like receptor G1 (KLRG-1), inducible T-cell co-stimulatory (ICOS), glucocorticoid-induced TNFR-related protein (GITR), chimeric antigen receptor (CAR), tumor-associated antigen (TAA).

Recognition of a tumor antigen through CAR-Ts triggers the activation of T-cells by providing a co-stimulatory signal. This, in turn, leads to the proliferation of CAR-Ts and the acquisition of effector functions [81]. CAR-Ts are equipped with either a CD28 or a 4-1BB co-stimulatory domain. However, there is ongoing exploration to assess the potential benefits of incorporating additional co-stimulatory molecules, such as CD27, ICOS, and OX40 [82]. The presence of a co-stimulatory signal plays a crucial role in maintaining the persistence and toxicity of CAR-Ts, influencing the effectiveness of this therapy against tumors [81,83], whereas co-inhibitory receptors such as programmed death-1 (PD-1), cytotoxic T lymphocyte antigen-4 (CTLA-4) (CD152), LAG-3 (CD223), T-cell immunoglobulin-3 (TIM-3), and TIGIT are crucial negative regulatory signaling pathways in T-cells [84]. Co-inhibitory receptors play a significant role in modulating T-cell responses and have demonstrated efficacy targets in the context of chronic diseases [21]. The important issue is preventing intrinsic dysfunctional pathways in CAR-Ts (e.g., inhibitory receptors signaling) and generating “exhaustion-resistant” cells [85]. PD-1 and CTLA-4 are cell surface receptors expressed by both CD4^+^ and CD8^+^ T-cells that function as T-cell checkpoints and play a central role in cancer immunotherapy [86,87]. TIM-3 is a type I transmembrane protein, serving as a distinctive marker for Th1 and Tc1 cells [88], whereas LAG-3 is expressed on activated CD4^+^ and CD8^+^ effector T-cells, CD4^+^Foxp3^+^ Treg, Tr1 cells, B cells, a subset of NK cells, and plasmacytoid DCs [89,90]. T-cell immunoglobulin and ITIM domain protein (TIGIT) is a type I transmembrane protein, affiliated with the immunoglobulin superfamily (IgSF), and it is expressed in both T-cells and NK cells [91] (Table 1). These receptors exhibit unique functions, particularly within tissue sites. They play pivotal role in regulating T-cell responses and upholding immune homeostasis [92,93,94,95]. It was confirmed that the increased expression of both co-stimulatory and co-inhibitory receptors on the surface of CAR-Ts is linked to the development of effector polyfunctional and exhausted hypofunctional phenotypes [96] (Figure 1).

## 7. Exhaustion and Senescence Markers in CAR-T Therapy

T-cell exhaustion and senescence have various common features, including defective effector functions, impaired proliferation, and cell cycle arrest [114]. The exhaustion of CAR-Ts results from continuous antigen stimulation and the presence of an immunosuppressive tumor microenvironment. Effectively addressing exhaustion is a critical challenge to sustain CAR-T effector function and persistence, aiming to achieve clinical potency [115]. Continual stimulation by antigen, the existence of inhibitory immune cells and cytokines in tumor microenvironment (TME), heightened expression of inhibitory receptors, alterations in T-cell-associated transcription factors, and metabolic factors can collectively lead to the T-cell exhaustion [116]. Exhausted T-cells are characterized as effector T-cells exhibiting reduced effector function, diminished cytokine expression, and a decreased responsiveness to reactivation [117]. Exhausted T-cells may highly express multiple “inhibitory” receptors, like PD-1, 2B4 (CD244), BTLA, CTLA-4, CD160, LAG-3, and TIM-3 [118,119,120]. The onset of CAR-T exhaustion is linked suppressive immune cells, including regulatory T-cells (Treg), myeloid-derived suppressor cells (MDSCs), tumor-associated macrophages (TAMs), cancer-associated fibroblasts (CAFs), tumor-associated neutrophils, and mast cells and to external inhibitory signals (such as TGF-β, IL-10, PGE2, soluble FAS, adenosine, ROS) [121]. Within the TME, tumor cells generate various suppressive mediators (i.e., PD-L1, TGF-β, IL-10, PGE2) to counteract efficient immune responses. The activation of negative signals within tumor cells can initiate immunosuppressive pathways, resulting in CAR-T dysfunction [122]. Importantly, tumor-derived EVs are active contributors to immunosuppression within the TME and promoting metastasis. These EVs have the capability to modify their phenotype and functions upon interaction with T-cells, initiating signaling through TCR or CAR and reprogramming them to evade immune response [123]. Chronic exposure of CD19-CAR-T to CD19+EVs triggers activation and systemic exhaustion in an antigen-specific manner, and this adverse impact is accompanied by impaired cytotoxic activity [124]. Importantly, the impairment of CAR-T function resulting from exhaustion is recognized as a pivotal factor contributing to treatment failure [123]. Also, senescence of T-cells plays an immunosuppressive role, particularly in aging individuals and cancer patients [125]. Cellular senescence is a multi-causal process that occurs in a variety of cell types and is characterized by cell cycle arrest [126]. Senescent T-cells tend to have a CD45RA^+^CD27^−^CD28^−^KLRG1^+^CD57^+^ phenotype and express cytolytic molecules, IFNγ, and TNF-α, but they lose their capacity for proliferation and their ability to release IL-2 [127].

## 8. Immunosuppressive Tumor Microenvironment in Hematological Malignancies

The effectiveness of CAR-T therapy and the risk of toxicities are significantly influenced by the immunosuppressive TME [14,128,129]. Overly suppressing immune responses within the TME facilitates the tumor progression [130]. It was confirmed that the effectiveness of CAR-T therapy against tumors with poor responsiveness can be boosted by co-administering the cells with inhibitors targeting immune checkpoint blockade [131]. Immune checkpoint receptors such as PD-1 and CTLA-4, expressed on activated T-cells, regulatory T-cells (Tregs) could preclude cytotoxicity of CAR-Ts and induce anergy within the TME [132]. Both chronic and acute leukemia elude immune system surveillance and instigate immunosuppression by amplifying preleukemic Foxp3^+^ Tregs. Elevated levels of these immunosuppressive Tregs are indicative of less favorable response to chemotherapy, increased likelihood of leukemia relapse, and shorter overall survival [133]. In ALL, the bone marrow microenvironment delivers signals for growth and survival that may confer resistance to chemotherapy and consequently contributing to the progression of B-ALL [134,135]. While the direct targeting of tumor cells is essential, it is equally crucial to overcome the immunosuppressive TME. The microenvironment in multiple myeloma (MM), leukemia, and lymphoma comprise components supportive of tumors, including stromal cells, myeloid-derived suppressor cells, regulatory T-cells, tumor-associated macrophages, and tumor-associated neutrophils [136]. These components interact closely with malignant cells, fostering their survival and facilitating immune evasion [137]. In addition, these immunosuppressive components diminish the cytotoxic impact of CAR-Ts, leading to exhaustion of CAR-Ts [138] (Table 2).

Notably, the toxicity and resistance mechanisms of CAR-T therapy are linked to the myeloid compartment. A new method for identifying patients with r/r large B-cell lymphoma at a very high risk of progression after CAR-T therapy involves assessing peripheral blood monocytes during leukapheresis. It makes it possible to evaluate CAR-Ts and highlights the necessity to include monocyte depletion strategies for better CAR-T production [16]. It was shown that early signs of neuroaxonal injury correlate with a higher proportion of senescence CD8^+^ T-cells and monocytic-myeloid derived suppressor cells (M-MDSC), which confirms that Immune Effector Cell-Associated Neurotoxicity Syndrome (ICANS) may be associated with pre-CAR-T systemic inflammation [17].

## 9. Cell-Free DNA as a Marker for MRD Monitoring

Cell-free DNA exists as fragmented pieces, with a predominant size peak at 166–167 base pairs, and circulating tumor tends to be shorter than regular cfDNA [146,147]. The relative amount of ctDNA within cfDNA can exhibit significant variability, spanning from 3% to 93% [148]. Usually, trace amounts of this cfDNA can be discerned in blood [149,150]. By leveraging advancements in DNA sequencing technologies, researchers are currently investigating cfDNA as a biomarker for identifying malignancies in their early stages, before symptoms appear [151]. cfDNA sequencing has demonstrated potential as a noninvasive diagnostic tool for assessing health, as well as for detecting cancer at an earlier stage and monitoring the response to treatment [152,153,154,155,156,157,158]. To precisely monitor therapeutic response in B-cell lymphoma patients receiving CAR-T therapy, multiple liquid biopsy technologies are used [159,160,161].

The analysis of cfDNA/ctDNA has the potential for diagnosing, predicting outcomes, and monitoring cancer [162]. However, it is crucial to note that cfDNA levels can also be elevated due to various other situations, including infection, trauma, inflammation, transplantation, and autoimmune conditions [163]. Research on cfDNA originating from lymphomas has suggested enhanced risk evaluation during the surveillance of minimal residual disease (MRD) [164,165]. Previous research has utilized next-generation sequencing (NGS) to analyze cellular samples from pediatric leukemia patients, providing insights into the mutational patterns at the time of diagnosis and relapse, as well as measuring immunoglobulin clonality as a sensitive indicator of remaining disease [161,166,167,168,169,170,171,172,173,174,175].

## 10. miRNAs as Markers in CAR-T Therapy

microRNAs (miRNAs) are short RNA molecules (21–23 nucleotides in length) that naturally occur within cells and regulate gene expression. miRNAs often function collectively, forming co-regulating groups participating in the same cellular processes [176]. These groups can be found in clusters of miRNAs, which may be transcribed as a single polycistronic transcript or consist of structurally unrelated miRNAs that are co-expressed and functionally associated. miRNAs play essential roles in regulating transcription, translation, and epigenetic processes. The coordinated action of clustered and co-expressed miRNAs can produce specific phenotypic effects, such as oncogenic or tumor suppressor effects [177]. It was shown that miR-146a could induce cytotoxic effects in leukemia cells in vitro and inhibit the expression of NF-κB target genes. This work suggests that miR-146a mimics targeted at myeloid cells may be used to treat myeloproliferative and inflammatory diseases [178]. Another work in which CAR-Ts artificially increased expression of miR-155 exhibited increased anti-tumor functions in vitro and in vivo [179]. However, to date, the issues related to miRNAs in hematological diseases have not been sufficiently studied, and all the information refers more to specific cancers such as NHL or ALL or in the context of T lymphocytes than to CAR-T therapy itself [180,181,182,183,184,185,186,187,188].

## 11. miRNAs as a Drug Resistance Marker

**Figure 2:**
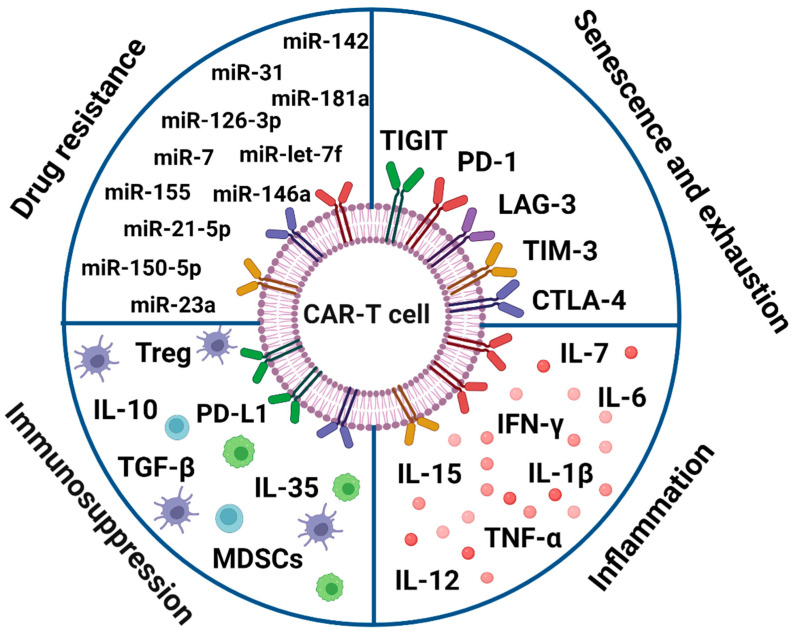
Biomarkers in CAR-T therapy. Programmed cell death protein 1 (PD-1), cytotoxic T-lymphocyte-associated protein-4 (CTLA-4), T-cell immunoglobulin and mucin domain 3 (TIM-3), T-cell immunoreceptor with immunoglobulin and ITIM domain (TIGIT), lymphocyte-activated gene 3 (LAG-3), chimeric antigen receptor T-cell (CAR-T), interleukin (IL), tumor growth factor β (TGF-β), myeloid-derived suppressor cells (MDSC), interferon γ (IFN-γ), T regulatory (Treg).

It has been shown that miRNAs have a significant role in the development of cancer, particularly hematological tumors, as well as in the disease’s aggressiveness, progression, and response to therapy. Furthermore, miRNAs have been closely linked to the alteration of cancer cells’ susceptibility to a variety of anticancer medications as well as cancer treatment resistance. Additionally, the function of miRNAs enclosed in extracellular vesicles (EVs-miRNAs) has been documented, and these EVs-miRNAs have been identified as critical for the horizontal transfer of drug resistance to susceptible cells. Numerous studies have proposed the use of miRNAs as promising therapeutic strategies in hematological illnesses and as biomarkers for medication response and clinical outcome prediction. In fact, overcoming drug resistance is facilitated by the combination of traditional medications with miRNA-based therapy techniques [189]. It has recently been discovered that hematologic malignancies, particularly ALL, exhibit dysregulated expression of several microRNAs (miRNAs). Further research is necessary to fully assess the function of cytomegalovirus infection in these domains, as it can cause ALL in otherwise healthy persons. MicroRNA expression in the plasma signature may serve as a potent diagnostic and prognostic marker, offering insights beyond cytogenetics. Given that CMV^+^ and post-HSCT GVHD patients had greater plasma levels of miR-92 and miR-155, elevating miR-155 in plasma may be a useful therapeutic target for ALL patients [190]. It was shown that miRNAs carried by EVs spread pro-senescence signals to endothelial cells, affecting DNA methylation and cell replication [191] (Figure 2).

## 12. EVs as Markers in CAR-T Therapy

**Figure 3:**
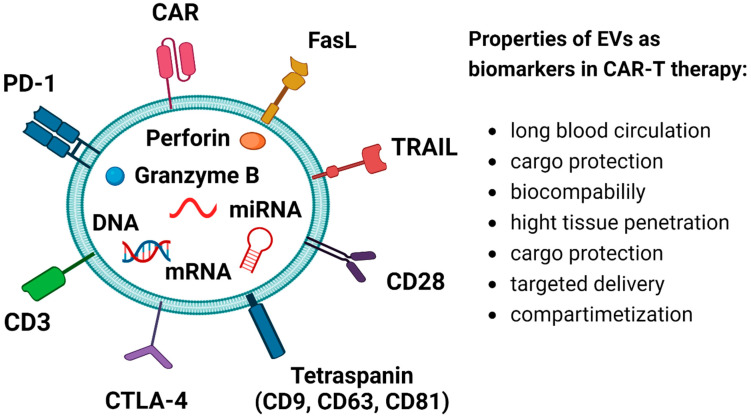
EVs as biomarkers in CAR-T therapy. Programmed cell death protein 1 (PD-1), chimeric antigen receptor (CAR), Fas ligand (FasL), TNF-related apoptosis-inducing ligand (TRAIL), cytotoxic T-lymphocyte-associated protein-4 (CTLA-4).

Extracellular vesicles (EVs) are vesicles derived from endosomes, ranging in diameter from 30 to 120 nm, and comprising nucleic acids (e.g., DNA, mRNA and non-coding RNAs (ncRNAs)), proteins, lipids, and metabolites [192,193,194,195,196,197,198]. Like cfDNA, EVs are also present in diverse bodily fluids, including peripheral blood, urine, saliva and cerebrospinal fluid [199,200]. EVs play crucial roles both normal and pathological conditions, contributing to the maintenance of cell homeostasis and regulation gene transcription [194]. The characteristics of EVs vary based on their cellular source, and the composition and expression of EVs released by healthy cells differ from those released by tumor cells. Hence, they hold promise as outstanding biomarkers for diagnosing, prognosis, and management of NHL and ALL patients at various stages [201]. Oxidative stress-altered intercellular communication, inflammation, genomic instability, epigenetic alterations, and stem cell exhaustion are associated with aging, whereas EVs could function as innovative biomarkers to capture the intricate nature of senescence [202]. It was confirmed that EVs released from senescent cells could stimulate the proliferation of cancer cells [203]. EVs transport surface molecules like checkpoint inhibitors and have potential to engage with CAR-sT, modifying their phenotype and functions by initiating signaling through TCR or CAR, consequently reprogramming them to evade the immune response [124]. Importantly, EVs originating from tumors actively contribute to metastasis and immunosuppression within the tumor microenvironment [204]. They can induce inappropriate cytokine release, leading to the exhaustion of CD19 CAR-Ts (Figure 3) [123]. Nucleic acids transported by EVs can establish an immunosuppressive environment for tumor cell growth. This occurs by promoting immunosuppressive cell populations such as myeloid-derived suppressor cells (MDSCs) and by inhibiting the anti-tumor immune responses of immune cells like DCs, NK cells, and T lymphocytes. This process facilitates immune evasion by tumors and enhances the metastasis of tumor cells to distant sites [205,206].

EVs and their cargo show potential as non-invasive indicators that can be used to monitor DLBCL patients after treatment. Biomarkers derived from EVs for DLBCL include miR-379-5p, miR-135a-3p, miR-146a, miR-124, and miR-532-5p. miR-15a-3p, miR-21-5p, and miR-181, miR-15a-3p, miR-21-5p, miR-181a-5p, and miR-4476 were found to be highly expressed in DLBCL patients compared to healthy individuals, while miR-483-3p, miR-425, miR-141, miR-145, miR-197, miR-345, miR-424, miR-128, miR-122, and miR-451a showed lower expression levels [195,207,208,209,210]. It was also observed that higher levels of miR-20a, miR-20b, miR-93, and miR-106a/106b in the plasma were associated with increased mortality rates [209]. Recent findings indicate that miRNAs, particularly miR-181b-5p, enriched in EVs from circulating leukemic cells, may function as valuable prognostic biomarkers for childhood ALL. Studies have demonstrated the involvement of miR-181b-5p in promoting leukemic cell proliferation, migration, and invasion [211].

## 13. EVs in Drug Resistance of Hematological Malignancies

EVs have an important role in communication between tumor cells and the TME [212,213]. It has been shown that they are also associated with drug resistance (DR) [204]. The enhanced proliferation of cancer cells leads to changes in oxygen levels, inducing hypoxia and prompting the release of exosomes by the cancer cells [214]. Over time, the majority of tumors develop resistant to various anticancer agents, even those chemically unrelated, following repeated treatment. The diminished accumulation of drugs in tumor cells is regarded as a significant mechanism, achieved by reducing drug permeability and/or increasing active efflux (pumping out) of drugs across the cell membrane [215]. A crucial aspect in the development of hematological malignancies involves elucidating the role of miRNAs, emphasizing their significant impact (whether in their cell-free circulating state or within circulating EVs) on drug resistance and cancer relapse, as well as their potential clinical applications. The detailed exploration of studies focusing on the involvement of miRNA from EVs in DR, along with their mechanism, is extensively discussed in the context of leukemia, lymphoma, and multiple myeloma [214,216]. It has also been shown that EVs could modulate the work of the macrophages, dendritic cells, T-cells, or NK cells, impacting TME [217].

Despite advancements in systemic cancer treatments, chemotherapy remains a cornerstone in the therapy of numerous cancer types. Nevertheless, the efficacy of chemotherapy is notably constrained by the partial or complete resistance of cancerous cells to cytotoxic drugs [218,219]. Presently, the literature underscores that extracellular vesicles (EVs) are key regulators of chemotherapy resistance, a phenomenon substantiated by various experimental and clinical studies. The composition of EVs may provide insights into the mechanisms underlying resistance to chemotherapy [219]. Feng et al. discovered that miR-99a-5p and miR-125b-5p expression levels in EVs circulating in the bloodstream were notably elevated in patients with chemoresistant DLBCL compared to well responders. Furthermore, they observed a connection between levels of exosomal miRNAs and shorter duration of progression-free survival, indicating their potential to predict the effectiveness of chemotherapy [195,220]. Increased concentration of exosomal miR-125b-5p and miR-99a-5p corelated with a shorter progression-free survival (PFS), whereas reduced expression of exosomal miRNA-107 and miR-451a indicated unfavorable prognosis in DLBCL [220,221]. Studies have demonstrated that miR-107 functions as a tumor suppressor by inhibiting oncogenes like FOXO1, PEPCK, CCND1, P27, BAD, and Bcl-2. Consequently, the downregulation of miR-107 is linked to shorter PFS in DLBCL [222]. Considering this pathway, miR-107 emerges as a promising therapeutic target in DLBCL [222].

## 14. Combination Immunotherapy with CAR-Ts, Checkpoint Blockade, and Other Drugs

Recent developments in the design and production of these agents have led to the development of more potent and less toxic monoclonal antibodies, bispecific T-cell engagers, and antibody–drug conjugates [19]. It has been confirmed that chimeric antigen receptor T-cell (CAR-T) therapy with monoclonal antibody (mAb)-based immune checkpoint blockade (ICB) is effective in hematologic malignancies [99]. From the initial lines of therapy to the relapsed and refractory setting for non-Hodgkin lymphoma (NHL), combination immunotherapy using CAR-T, checkpoint inhibitors, and monoclonal antibodies is being incorporated into lymphoma treatment [223]. Combining PD-1 inhibitors with CD19 CAR-T therapy has enhanced clinical outcomes in B-ALL patients. CD19-targeted CAR-Ts induce long-lasting remissions in approximately 30% to 40% of r/r large B-cell lymphomas. CAR-T failure can result from T-cell exhaustion or an immunosuppressive tumor microenvironment. Pembrolizumab, an anti-PD1 immune checkpoint inhibitor, may alleviate T-cell exhaustion following CAR-T therapy [97]. Additionally, PD-1 blockade therapy can be effective in patients with r/r DLBCL after failure of CAR-T therapy who had PD-L1 expression in tumor cells and high PD-1 levels in tumor-infiltrated T-cells [98]. CD19^−^PD-1/CD28^−^CAR-Ts, an innovative anti-CD19 CAR-T therapy, induce a strong and lasting anticancer response and can be employed after CD19-CAR-T failure [224].

**Figure 4:**
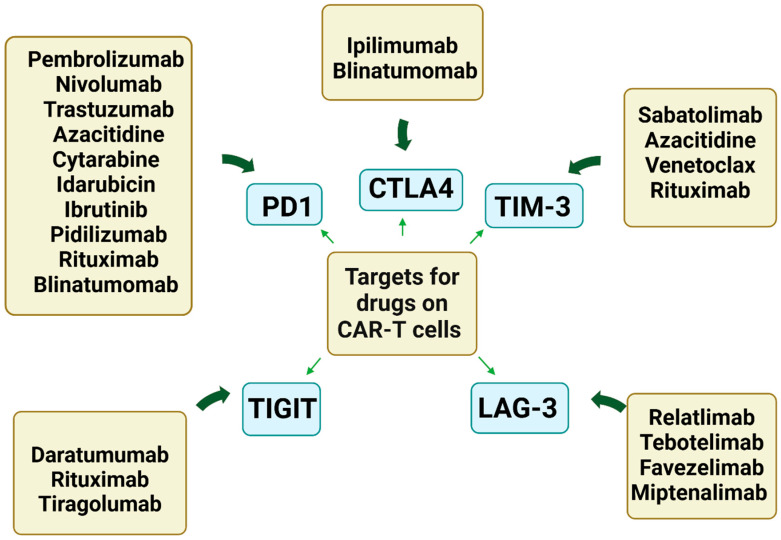
The drugs target co-inhibitory molecules that regulate the exhaustion and senescence of CAR-Ts. Programmed cell death protein 1 (PD1), cytotoxic T-lymphocyte-associated protein-4 (CTLA4), T-cell immunoglobulin and mucin domain 3 (TIM-3), T-cell immunoreceptor with immunoglobulin and ITIM domain (TIGIT), lymphocyte-activated gene 3 (LAG-3).

Bispecific T-cell engagers (BiTEs) such as blinatumomab, were approved by the U.S. Food and Drug Administration (FDA) for use in multiple B-cell malignancies. BiTE therapy is used in combating minimal (or measurable) residual disease in patients with acute lymphoblastic leukemia [225]. Blinatumomab (Blincyto, Amgen) is approved for r/r B cell precursor acute lymphoblastic leukemia (B-ALL) and B-cell precursor ALL with MRD [226]. It is also being studied in combination with other therapies, such as tyrosine kinase inhibitors, checkpoint inhibitors, and chemotherapy, across various treatment settings, including frontline protocols [109,227]. Bispecific CAR-Ts targeting both CD19 and CD22 have emerged as effective treatment options for chemoresistant B-ALL [227,228]. Bispecific antibodies (BsAbs) CD20 × CD3 including odronextamab, mosunetuzumab, and glofitamab have promising efficacy in r/r NHL with favorable toxicity profiles and reduced cytokine release syndrome and neurotoxicity [229]. It was shown that bispecific CARs targeting CD20/CD19, incorporating 4-1BB and mut06 co-stimulation, are associated with antitumor activity, increased persistence, and decreased exhaustion [230]. Furthermore, cytotoxic drugs have an impact on proliferation, survival, and blasting T-cells. Pre-treatment with regimens containing cyclophosphamide and doxorubicin seems to be linked to underperforming CAR-Ts, possibly indicating cellular senescence (Figure 4) [231].

## 15. Conclusions and Future Perspectives

One of the most promising methods for managing cancer is personalized immuno-oncology, but it also has some limitations caused by immunosuppressive metabolites, defective antigen presentation, or a lack of response-predictive biomarkers. To overcome these problems, future methods should consider the immunosuppressive microenvironment and inhibitory potential of natural immune cells. The potential promising cytotoxic cells for the therapy of hematological malignancies include armored CAR-Ts. Combining CAR-T therapy with other medications or checkpoint inhibitors seems like a promising approach. Additionally, chemoresistant patients now have excellent therapy alternatives in the form of bispecific T-cell engagers. Accurately identifying patients to assess the overall risk of secondary primary malignancy after CAR-T therapy is essential for optimal cancer treatment [232,233]. Patients who respond rapidly to initial treatment may benefit from shorter treatment regimens. Also, the involvement of miRNAs in cancer is associated with prognostic implications. Circulating miRNAs hold promise in aiding clinical decision-making as they exhibit high stability in blood samples. Biomarkers are crucial for toxicity, efficacy forecasting, and relapse evaluation. They can also be used in clinical CAR-T therapy applications and to create safe and effective personalized medication. A growing number of researches are examining different biomarkers that can forecast their efficacy and potential for toxicity. Importantly, CAR-T therapy has the potential to revolutionize cancer treatment and improve outcomes for patients with solid tumors, including glioblastoma, especially when combined with specific targeted drugs [234]. However, further research is required to fully comprehend the special side effects of immunomodulation, to ascertain the best order and combination of this medication with conventional chemotherapy and targeted therapies, and to find reliable predictive biomarkers. Future advances regarding markers are necessary to increase diagnostic sensitivity in clinical procedures.

## Figures and Tables

**Table 1:** Co-inhibitory receptors involved in the regulation of exhaustion and senescence of CAR-Ts in combination with immune checkpoint inhibitors and other drugs.

Receptor	Exhaustion/Senescence	Combination with Immune Checkpoint Inhibitors and Other Drugs	CAR-T Study in Hematological Malignancies [References]
PD-1	Exhaustion	Pembrolizumab, Nivolumab, Trastuzumab, Azacitidine, Cytarabine, Idarubicin, Ibrutinib, Pidilizumab, Rituximab, Blinatumomab	[97,98,99,100,101,102,103,104,105,106,107,108,109]
CTLA-4	Exhaustion	Ipilimumab, Blinatumomab	[99,107,109,110]
TIM-3	Exhaustion/Senescence	Sabatolimab (MBG453), Azacitidine, Venetoclax, Rituximab	[99,107,111]
LAG-3	Exhaustion/Senescence	Relatlimab (BMS-986016), Favezelimab (MK-4280), Miptenalimab (BI754111), Tebotelimab (MGD013)	[99,107,112] https://clinicaltrials.gov/ (accessed date 11 July 2024)
TIGIT	Exhaustion/Senescence	Tiragolumab (MTIG7192A, RG6058), Daratumumab, Rituximab	[99,108,113] https://clinicaltrials.gov/ (accessed date 11 July 2024)

**Table 2:** Immunosuppressive function of immune checkpoint receptors.

Receptor	Immunosuppressive Function	References
PD-1	regulates T-cell response controls tissue damage caused by the immune system resolves inflammation by adjusting the intensity and duration of immune response induces T-cells to enter a state of exhaustion, tolerance, or dysfunction	[129,139,140,141]
CTLA-4	exerts a suppressive signal on T-cells makes T-cells with an inactive state boosts Treg activity enhances IDO and IL-10 production in DCs essential regulator of T-cell homeostasis and self-tolerance	[139,142,143]
LAG-3	impairs CD4^+^ and CD8^+^ TILs functions inhibitory receptor and exhaustion marker serves crucial function in autoimmune response, tumor immunity, and defense against infection	[21,92,139,143]
TIM-3	suppresses activation and activity of CTLs stimulates apoptosis of immune cells	[21,92,144]
TIGIT	regulates T-cell function maintains self-tolerance controls active T-cell responses at peripheral tissues	[21,92,145]
